# Multimodal Treatment Approach for a Recurrent Hemorrhagic Scalp Arteriovenous Fistula: A Case Report

**DOI:** 10.7759/cureus.74278

**Published:** 2024-11-22

**Authors:** Junpei Kato, Tatsuya Tanaka, Fumitaka Yamane, Masaaki Akahane, Yuichiro Hayashi, Akira Matsuno

**Affiliations:** 1 Department of Neurosurgery, International University of Health and Welfare Narita Hospital, Narita, JPN; 2 Department of Neurosurgery, International University of Health and Welfare Atami Hospital, Atami, JPN; 3 Department of Radiology, International University of Health and Welfare Narita Hospital, Narita, JPN; 4 Department of Anatomic Pathology, International University of Health and Welfare Narita Hospital, Narita, JPN

**Keywords:** complications, endovascular embolization, infective endocarditis, scalp arteriovenous fistula, skin grafting, surgical excision, vascular malformation

## Abstract

Scalp arteriovenous fistula (AVF) is a rare vascular malformation that may present as a pulsatile scalp mass with complications like hemorrhage. We report a case of a large scalp AVF with recurrent bleeding, managed successfully with a multimodal approach. A 46-year-old man presented with a recurrently bleeding pulsatile scalp mass in the left temporal region, initially diagnosed as AVF following trauma. Imaging revealed an 80-mm lesion, and staged treatment included embolization, direct n-butyl-2-cyanoacrylate (NBCA) injection, resection, and skin grafting. Postoperative infective endocarditis required mitral valve replacement and prolonged antibiotics. At one-year follow-up, no AVF recurrence was observed. Scalp AVFs can lead to serious complications if untreated. This case demonstrates successful management through embolization and excision, highlighting the importance of individualized treatment strategies based on lesion complexity.

## Introduction

Scalp arteriovenous fistula (AVF) is a rare vascular malformation characterized by direct connections between feeding arteries and draining veins within the subcutaneous tissue [1-4]. Clinically, patients often present with a pulsatile scalp mass, accompanied at times by symptoms such as headache or tinnitus [1-4]. Complications such as hemorrhage, infection, and skin necrosis can arise as the overlying skin becomes atrophic [1-4]. Here, we present a case of a large scalp AVF that progressively enlarged, resulting in recurrent hemorrhage. This condition was successfully managed with a multimodal approach involving embolization, surgical excision, and skin grafting.

## Case presentation

**Figure 1:**
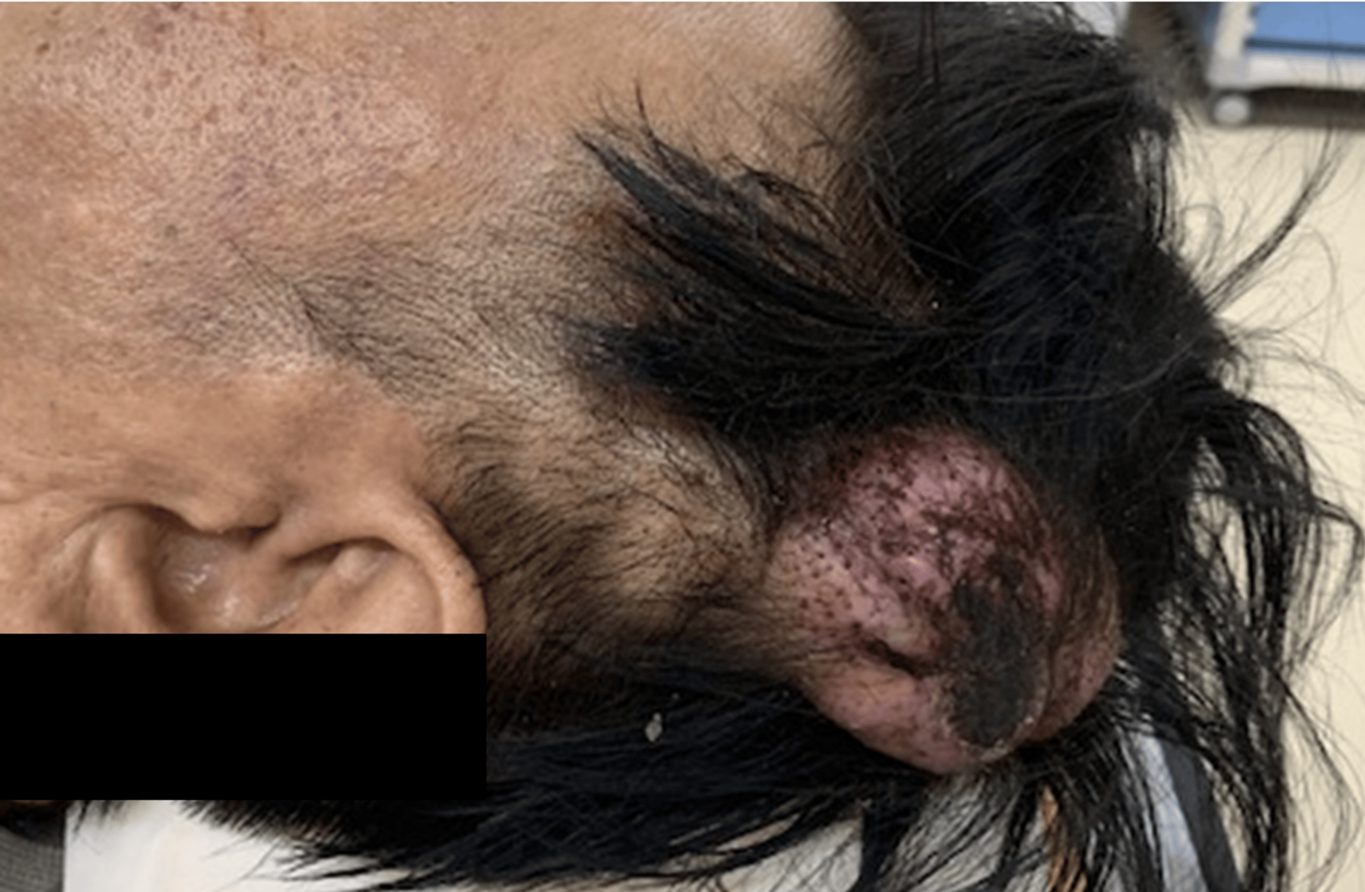
Clinical presentation A pulsatile mass with dilated vessels, hemorrhagic spots, and crust formation is observed in the left temporal region.

A 46-year-old man presented with a recurrently bleeding large pulsatile mass in the left temporal region. His medical history included idiopathic portal hypertension and gastric varices. At age 28, he sustained a head injury in the left temporal region during a traffic accident, requiring three stitches. Over the ensuing years, a pulsatile mass gradually developed at the injury site, and at age 33, he was diagnosed with scalp AVF and treated with sclerotherapy using absolute ethanol. Although the mass temporarily regressed, it recurred the following year. By age 46, he began experiencing spontaneous bleeding episodes from the lesion. Physical examination revealed an 80-mm, soft, pulsatile mass with black crusting in the left temporal region (Figure 1).

**Figure 2:**
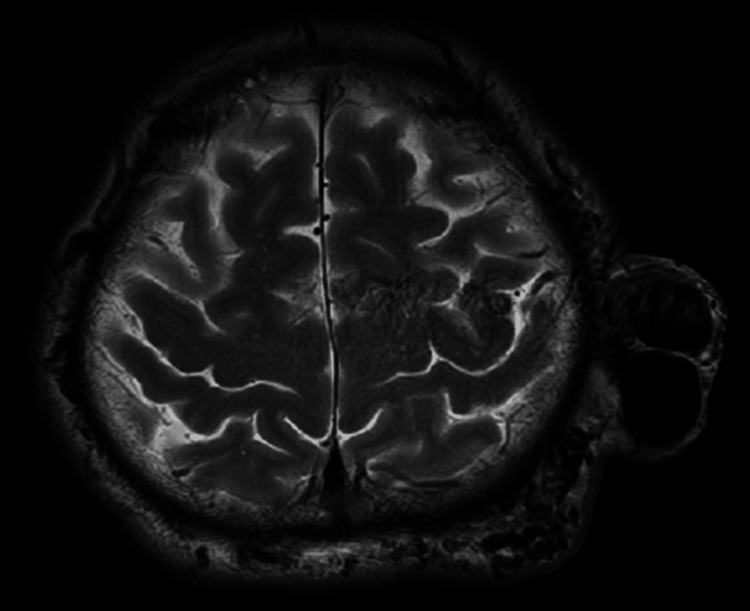
Initial head MRI T2-weighted MRI reveals a vascular tumor-like lesion in the subcutaneous tissue, measuring up to 80 mm in maximum diameter. MRI: magnetic resonance imaging

A bruit was auscultated, but cardiovascular and neurological examinations were unremarkable. Magnetic resonance imaging (MRI) revealed an 80-mm vascular lesion beneath the subcutaneous fat (Figure 2).

**Figure 3:**
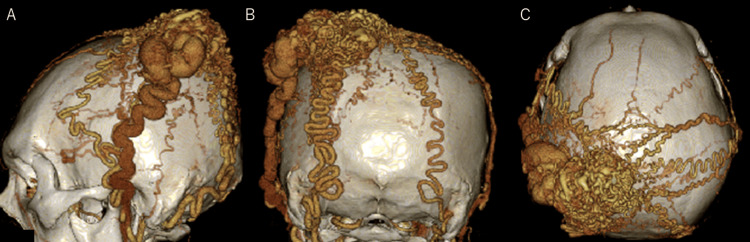
Preoperative 3DCTA (A: left lateral view, B: posterior view, C: superior view) 3DCTA demonstrates a nidus-like vascular cluster in the left temporal region. The feeding arteries are the bilateral superficial temporal arteries and the occipital artery, while the draining vein is the left superficial temporal vein. 3DCTA: three-dimensional computed tomography angiography

Computed tomography angiography (CTA) demonstrated tortuous dilated vessels (Figure 3).

**Figure 4:**
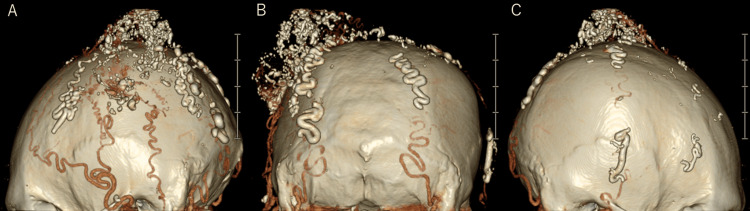
Post-embolization 3DCTA (A: left lateral view, B: posterior view, C: right lateral view) Post-embolization 3DCTA shows NBCA filling within the feeding arteries and the nidus. 3DCTA: three-dimensional computed tomography angiography, NBCA: n-butyl-2-cyanoacrylate

Angiography identified feeding arteries from the occipital arteries (OA) and superficial temporal arteries (STA) with drainage into the left superficial temporal vein (STV). Due to the progressive enlargement and recurrent hemorrhage, a staged intervention was planned. First, endovascular embolization was performed to reduce arterial supply to the mass. On Day 1, embolization of the left STA and the left OA was conducted. On Day 14, embolization of the right STA and the right OA was performed. A 6-French catheter was introduced via the right femoral artery and directed to the external carotid artery, followed by microcatheter access to the bilateral STA and distal OA. Embolization with n-butyl-2-cyanoacrylate (NBCA) was completed (Figure 4).

**Figure 5:**
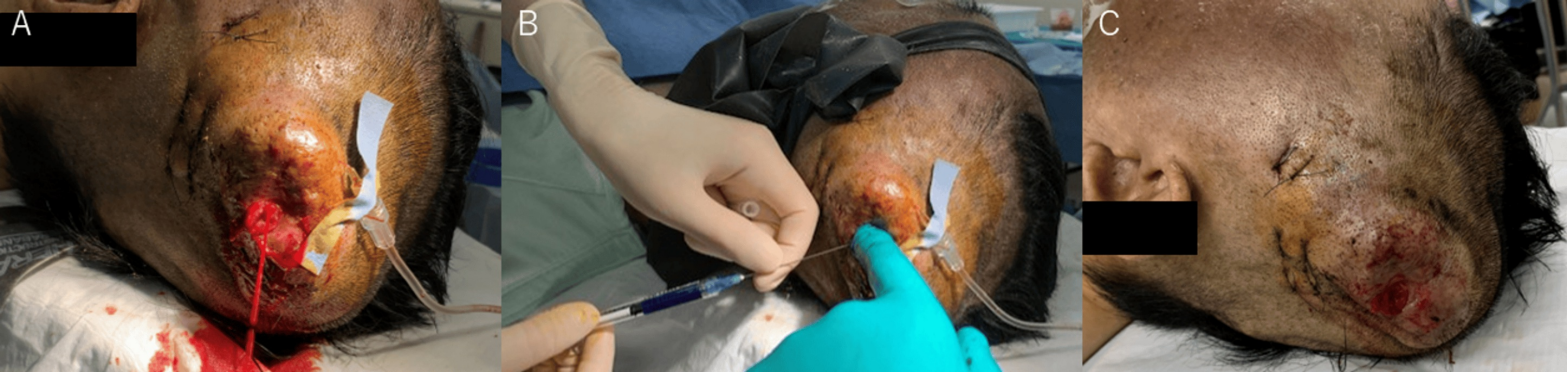
Direct puncture injection of NBCA A: Active arterial bleeding from the mass is noted. B: NBCA is directly injected into the mass via puncture. C: Hemostasis is achieved following NBCA injection. NBCA: n-butyl-2-cyanoacrylate

However, rebleeding occurred from the thin skin. On Day 43, additional interventions were undertaken, including applying a tourniquet to the scalp and directly injecting NBCA into the mass via puncture, achieving hemostasis (Figure 5).

**Figure 6:**
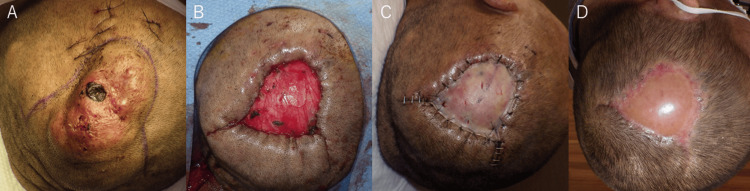
Mass resection and skin grafting A: Preoperative photograph showing the skin defect over the mass. B: Post-resection photograph illustrating the skin defect after mass excision. C: Split-thickness skin grafting is performed using a graft harvested from the thigh. D: At three months postoperative, the grafted skin shows good adherence and healing.

On Day 50, surgical excision and skin grafting were performed under general anesthesia (Figure 6).

**Figure 7:**
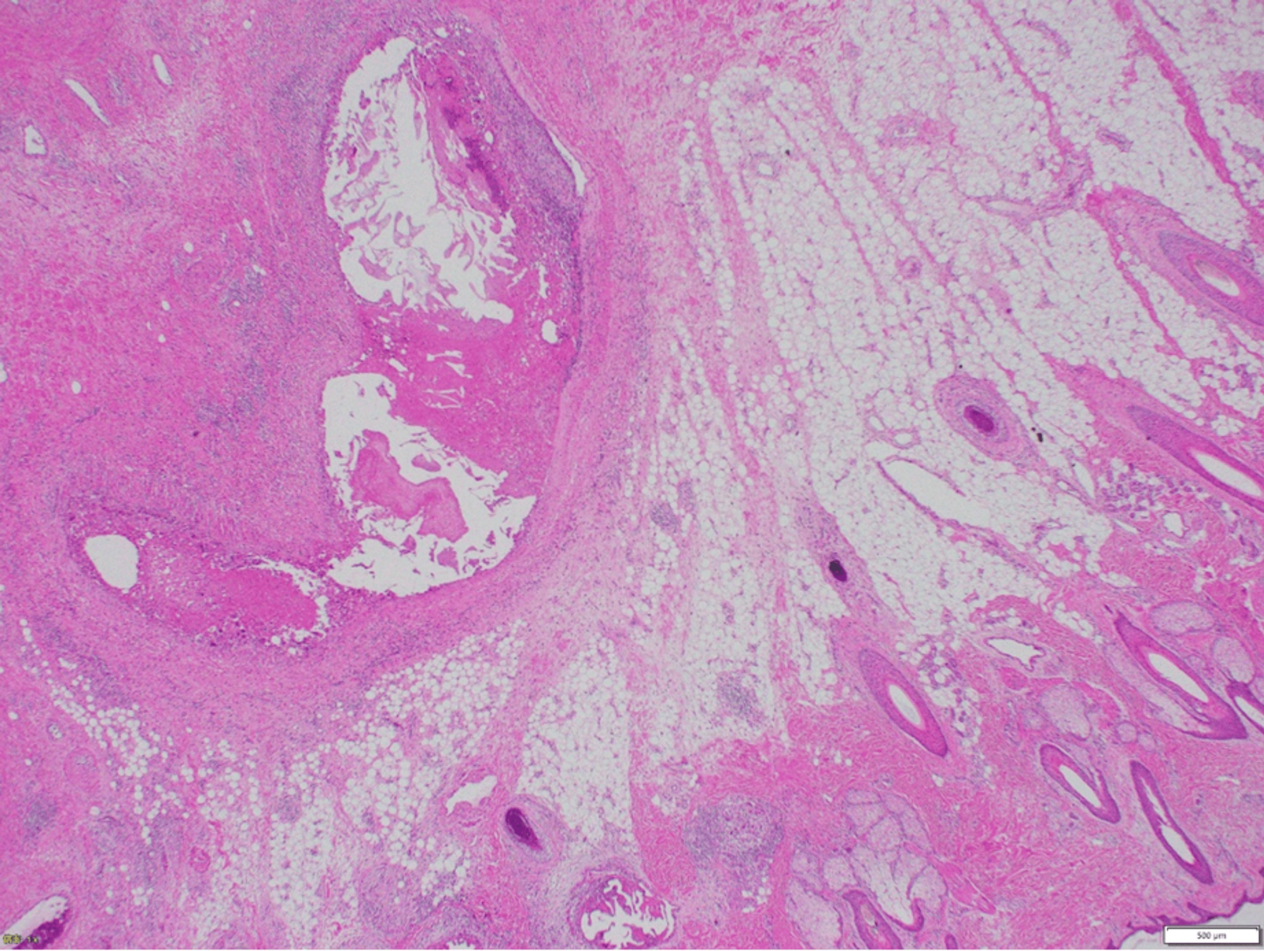
Histopathological finding Microscopic examination of the resected specimen (hematoxylin and eosin stain) reveals dilated vessels in the subcutaneous adipose tissue, with multiple arteries and veins present, consistent with an AVF. AVF: arteriovenous fistula

The skin incisions were designed to facilitate closure by approximating the skin from the parietal region after excision of areas of skin thinning and defects (Figure 6A). Feeding vessels were coagulated and cut sequentially: facial, parietal, occipital, and finally caudal, with the left STV, which serves as the main draining vein, ligated last. The mass was excised en bloc (Figure 6B). A split-thickness skin graft was harvested from the thigh (Figure 6C), with a successful graft take (Figure 6D). The surgery lasted three hours and 40 minutes, with a blood loss of 382 ml. Pathology confirmed the diagnosis of an AVF with numerous dilated arterial and venous vessels in the subcutaneous tissue (Figure 7).

On postoperative Day 10, the patient developed a fever. Blood cultures grew Staphylococcus aureus, and transthoracic echocardiography revealed moderate mitral regurgitation with a 3-cm mobile vegetation, leading to a diagnosis of infective endocarditis. The patient subsequently underwent mitral valve replacement and completed a prolonged course of antibiotics. He was discharged on postoperative Day 107. At the one-year follow-up, there was no recurrence of the AVF or the temporal mass.

## Discussion

Scalp AVFs are vascular lesions that develop within the subcutaneous tissue of the scalp, characterized by abnormal arteriovenous shunting and vascular proliferation [1-4]. Due to high blood flow, these lesions often exhibit tortuosity, dilation, and progressive enlargement and are associated with symptoms such as pulsatile masses, headache, tinnitus, hemorrhage, skin necrosis, and even congestive heart failure [1-4]. Etiological factors include idiopathic, trauma, and iatrogenic causes, such as hair transplantation and craniotomy [1-4]. In this particular case, the fistula originated from trauma, with hemorrhage and skin necrosis as prominent symptoms.

Various treatment strategies are available for scalp AVFs, including embolization, surgical excision, or a combination of both [1-4]. Embolic agents frequently used include coils, absolute ethanol, NBCA, and Onyx [5-8]. Recurrence is frequently observed following incomplete embolization, highlighting the importance of complete excision for preventing relapse [1-4]. Extensive resection may be required for large lesions, sometimes necessitating scalp reconstruction [1-4]. Preoperative embolization can be beneficial in reducing intraoperative blood loss and is recommended, especially for extensive lesions with multiple feeding vessels [2-4]. Multiple access routes can be used for embolization, such as percutaneous catheterization via the femoral artery or vein, catheterization of inflow and outflow vessels, or direct puncture of the lesion itself [5-8]. For larger, complex fistulas, preoperative endovascular embolization combined with surgical excision can reduce the extent of required scalp reconstruction. In this case, repeated episodes of arterial hemorrhage necessitated preoperative embolization before excision. In this case, repeated arterial hemorrhage necessitated preoperative embolization before surgical excision. Given the recurrent bleeding, liquid embolic agents were considered more suitable than coils. Among the embolic agents approved for use in Japan for scalp AVFs, only NBCA is available; thus, NBCA was utilized.

According to systematic reviews, the optimal treatment approach should be individualized based on lesion size and the number of feeding vessels [3]. Here, given that the lesion in this patient measured over 4 cm in diameter and had multiple feeding vessels, combined therapy with embolization and excision was deemed appropriate.

Treatment-related complications differ between excision and embolization. Surgical excision may lead to skin necrosis, infection, hemorrhage, sepsis, and skin pain [3], while embolization can result in residual masses, skin inflammation, skin necrosis, and multiple aneurysms [3]. In cases of skin necrosis, complete lesion removal and scalp reconstruction using rotational flaps or skin grafts may be necessary. In this patient, skin necrosis required a skin graft. Postoperative complications included sepsis and infective endocarditis, which necessitated mitral valve replacement and extended antibiotic therapy. The combination of repeated endovascular treatments, tumor resection, and skin grafting for necrotic tissue likely contributed to these infectious complications.

## Conclusions

Scalp AVFs are rare but potentially serious vascular lesions. Delayed diagnosis can lead to significant complications. In this case, a traumatic scalp AVF was successfully treated through a combination of endovascular embolization and surgical excision. Although the patient developed infective endocarditis postoperatively, a favorable outcome was achieved following mitral valve replacement and prolonged antibiotic therapy. An individualized treatment strategy, considering lesion size and vascular complexity, is crucial for the effective management of scalp AVFs.
